# Matrix metalloproteinase 12 is induced by heterogeneous nuclear ribonucleoprotein K and promotes migration and invasion in nasopharyngeal carcinoma

**DOI:** 10.1186/1471-2407-14-348

**Published:** 2014-05-20

**Authors:** I-Che Chung, Lih-Chyang Chen, An-Ko Chung, Mei Chao, Hsin-Yi Huang, Chuen Hsueh, Ngan-Ming Tsang, Kai-Ping Chang, Ying Liang, Hsin-Pai Li, Yu-Sun Chang

**Affiliations:** 1 Molecular Medicine Research Center, Chang Gung University, 259 Wen-Hwa Ist Road, Taoyuan, Kwei-shan 333, Taiwan; 2 Department of Medicine, Mackay Medical College, No.46, Sec.3, Zhong-Zheng Rd, New Taipei City, San-Zhi Dist 252, Taiwan; 3 Graduate Institute of Biomedical Sciences, Chang Gung University, 259 Wen-Hwa Ist Road, Taoyuan, Kwei-shan 333, Taiwan; 4 Department of Microbiology and Immunology, Chang Gung University, 259 Wen-Hwa Ist Road, Taoyuan, Kwei-shan 333, Taiwan; 5 Departments of Pathology, Chang Gung Memorial Hospital at Lin-Kou, 5, Fu-Ching St, Taoyuan, Kwei-Shan 333, Taiwan; 6 Departments of Radiation Oncology, Chang Gung Memorial Hospital at Lin-Kou, 5, Fu-Ching St, Taoyuan, Kwei-Shan 333, Taiwan; 7 Departments of Otolaryngology, Chang Gung Memorial Hospital at Lin-Kou, 5, Fu-Ching St, Taoyuan, Kwei-Shan 333, Taiwan

**Keywords:** hnRNP K, MMP12, Migration, Invasion, Nasopharyngeal carcinoma

## Abstract

**Background:**

Overexpression of heterogeneous nuclear ribonucleoprotein K (hnRNP K), a DNA/RNA binding protein, is associated with metastasis in nasopharyngeal carcinoma (NPC). However, the mechanisms underlying hnRNP K-mediated metastasis is unclear. The aim of the present study was to determine the role of matrix metalloproteinase (MMP) in hnRNP K-mediated metastasis in NPC.

**Methods:**

We studied hnRNP K-regulated MMPs by analyzing the expression profiles of MMP family genes in NPC tissues and hnRNP K-knockdown NPC cells using Affymetrix microarray analysis and quantitative RT-PCR. The association of hnRNP K and MMP12 expression in 82 clinically proven NPC cases was determined by immunohistochemical analysis. The hnRNP K-mediated MMP12 regulation was determined by zymography and Western blot, as well as by promoter, DNA pull-down and chromatin immunoprecipitation (ChIP) assays. The functional role of MMP12 in cell migration and invasion was demonstrated by MMP12-knockdown and the treatment of MMP12-specific inhibitor, PF-356231.

**Results:**

MMP12 was overexpressed in NPC tissues, and this high level of expression was significantly correlated with high-level expression of hnRNP K (P = 0.026). The levels of mRNA, protein and enzyme activity of MMP12 were reduced in hnRNP K-knockdown NPC cells. HnRNP K interacting with the region spanning −42 to −33 bp of the transcription start site triggered transcriptional activation of the MMP12 promoter. Furthermore, inhibiting MMP12 by MMP12 knockdown and MMP12-specific inhibitor, PF-356231, significantly reduced the migration and invasion of NPC cells.

**Conclusions:**

Overexpression of MMP12 was significantly correlated with hnRNP K in NPC tissues. HnRNP K can induce MMP12 expression and enzyme activity through activating MMP12 promoter, which promotes cell migration and invasion in NPC cells. *In vitro* experiments suggest that NPC metastasis with high MMP12 expression may be treated with PF-356231. HnRNP K and MMP12 may be potential therapeutic markers for NPC, but additional validation studies are warranted.

## Background

Heterogeneous nuclear ribonucleoprotein K (hnRNP K), a member of the hnRNP family, is aberrantly increased in multiple types of cancer [1-5], including nasopharyngeal carcinoma (NPC) [5]. HnRNP K is a nucleocytoplasmic shuttling protein that is primarily located in the nucleus, where it is involved in transcriptional regulation [6]. It may act as either a transactivator [7-9] or transrepressor [10], depending on the interacting factors involved. In terms of the post-transcriptional regulation of hnRNP K, its cytoplasmic accumulation is governed by ERK-mediated phosphorylation at serines-284 and −353 [11,12]. The tumorigenic potential of hnRNP K is mediated by various tumor-associated genes, such as FLIP [7], TP [11], eIF4E [13] and c-Myc [14]. High-level hnRNP K expression has been correlated with decreased metastasis-free survival in NPC patients and may promote metastasis of NPC cells in part by inducing downstream metastasis-related genes [5,7]. To investigate the regulatory mechanism underlying hnRNP K-mediated metastasis, microarray analysis were performed in the hnRNP K-knockdown or in control NPC cells. Our preliminary data indicated that matrix metalloproteinase 12 (MMP12) was one of the hnRNP K-activated downstream targets.

The MMP family has 23 members that differ in their substrate specificities toward various components of the extracellular matrix (ECM). Structurally, the MMPs generally include a highly conserved propeptide domain, a zinc-binding catalytic domain, and a hemopexin-like domain; a catalytic zinc ion is required for their proteolytic activity [15]. MMPs are involved in many phases of cancer progression, including tumor invasion, metastasis, and angiogenesis [16,17]. Previously, it has been reported that induction of MMP1 [18], MMP2 [19,20] and MMP9 [21] expression were detected and correlated with poor prognosis in NPC due to the invasive and metastatic role of MMPs. This increase in MMPs expression is mainly caused by EBV latent membrane protein 1 (LMP1) [22], LMP2A [23] and Zta [24]. To data, however, no study has specifically examined the expression of MMP12 in NPC. MMP12, also known as macrophage metalloelastase is overexpressed in many cancer types, and high-level MMP12 expression has been associated with poor prognosis and increased risk of metastasis in cancer patients [25-28]. In malignant cells, the tumor microenvironment, which contains various inflammatory mediators (e.g., TNFα and GM-CSF), was found to positively regulate MMP12 expression through the activation of NF-κB and AP-1 [29,30]. MMP12 has also been shown to be involved in cell invasion [31,32], proliferation [33] and angiogenesis [34].

NPC is more prominent in Southeastern China and Taiwan than in Western countries. Epidemiological studies have indicated that infection with Epstein-Barr virus (EBV), dietary habits, and genetic susceptibility might be critical cofactors in the development of NPC [35,36]. Radiotherapy is traditionally the first choice for treating primary NPC. Under the current combined treatments with both radio- and chemotherapy regimens, the survival rates among NPC patients are ~92% at 1 year and ~50% at 5 years, with 20-25% of patients eventually developing distant metastases [37].

We previously reported that hnRNP K can be a prognostic biomarker for NPC, and regulates TP and FLIP post-transcriptionally and transcriptionally, respectively [7,11]. In the present study, we show that hnRNP K can regulate MMP12 expression transcriptionally, and promotes the migration and invasion of NPC cells. MMP12 inhibitor PF-356231 prevents NPC cell migration and invasion *in vitro*. Clinically, elevated expression of MMP12 was significantly correlated with high-level expression of hnRNP K in NPC biopsy tissues.

## Methods

### Cell culture

The NPC-derived cell line, TW02 [38], derived from a keratinizing squamous cell carcinoma [World Health Organization (WHO) type I], was provided by Dr. C. T. Lin (National Taiwan University, Taiwan). The NPC-derived cell line, HK1 [39], derived from a keratinizing squamous cell carcinoma (WHO type I), was provided by Dr. S. W. Tsao (Hong Kong University, SAR, China). NPC-TW02 and NPC-HK1 cells were culture in Dulbecco’s modified Eagle’s medium (DMEM, Gibco BRL, Rockville, MD) and RPMI1640 (Gibco BRL), respectively. All NPC cell lines were supplemented with 10% fetal calf serum (FCS), 100 U/ml penicillin, and 100 μg/ml streptomycin at 37°C under 5% CO_2_.

### Affymetrix microarray analysis

RNA samples from hnRNP K-knockdown NPC-TW02 cells, control NPC-TW02 cells, nine individual NPC tissues and one pool of the corresponding adjacent non-tumor tissues, were isolated using the TRIzol reagent (Invitrogen, Carlsbad, CA). The biotinylated oligonucleotide was hybridized to the Human Genome U133 Plus 2.0 Array (Affymetrix, Santa Clara, CA) by the National Yang-Ming University Genomics Center (Taiwan), following the manufacturer’s instructions. Microarray data were analyzed using the GeneSpring software version GX 7.31 (Silicon Genetics, Redwood, CA). The ratio of the average hybridization intensity between hnRNP K-knockdown/control NPC-TW02 cells or NPC tumor/normal tissue was taken as the relative gene expression level.

### Quantitative RT-PCR

RNA samples from NPC-TW02, and -HK1 cells and NPC tissues were isolated using the TRIzol reagent (Invitrogen). Reverse transcription of RNA (1 μg) was performed using oligo(dT)_20_ primers (Invitrogen) and Moloney Murine Leukemia Virus (M-MLV) Reverse Transcriptase (Invitrogen) according to the manufacturer’s instructions. The primers used to amplify the cDNA corresponding to *MMP1*, *MMP12*, *MMP13*, *MMP28* and *GAPDH* are presented in Additional file 1: Table S1. Quantitative RT-PCR was performed on a Light-Cycler (Roche Diagnostics, Mannheim, Germany), using the FastStart DNA Master SYBR Green I reagent (Roche Diagnostics). The gene expression results were normalized with regard to the expression of the *GADPH*. For mRNA half-life assessment, actinomycin D (5 μg/ml) was added 48 hours after cells were transfected with control or hnRNP K-targeting siRNA, and RNA was prepared at the indicated times.

### RNA interference

*SMART* pool reagents, including four RNA duplexes targeting hnRNP K and MMP-12 were purchased from Dharmacon (Lafayette, CO), and the negative control siRNA was synthesized by Eurogentec S.A. (Liege, Belgium). The target sequences of the siRNA were as follows: hnRNP K, 5'-UAAAC GCCCU GCAGA AGAUU U-3', 5'-GGUCG UGGCU CAUAU GGUGU U-3', 5'-UGACA GAGUU GUUCU UAUUU U-3' and 5'-GCAAG AAUAU UAAGG CUCUU U-3'. NPC cells were transfected with double-stranded (ds) RNA duplexes (50 nmol/L) using the Lipofectamine 2000 reagent (Invitrogen).

### Patients and clinical characteristics

The retrospective cohort comprised 82 NPC patients who had been admitted to Chang Gung Memorial Hospital (Lin-Kou, Taiwan) from 1990 to 1998. Clinical stage was defined according to the 2002 cancer staging system revised by the American Joint Committee on Cancer. The study population included 17 stage-I-II and 65 stage-III-IV patients comprising 61 men and 21 women ranging from 22 to 78 years of age (median age 44). Histological typing was done according to the WHO classification criteria, as previously described [40]. This study was reviewed and approved by the institutional review board and ethics committee of Chang Gung Memorial Hospital. Informed consent was obtained from all patients.

### Immunohistochemical staining

Immunohistochemical analyses were performed as described previously [5,7,37,40], using an automatic IHC-staining device (Bond-max Automated Immunostainer; Vision Biosystems, Melbourne, Australia), according to the manufacturer’s instructions. Tissue sections were retrieved using Bond Epitope Retrieval Solution 1 (Vision BioSystems) and stained with antibodies against hnRNP K (mouse monoclonal antibody, 1:300 dilution; Santa Cruz Biotechnology, Santa Cruz, CA, USA) and MMP-12 (goat polyclonal antibody, 1:10 dilution; Santa Cruz Biotechnology). A polymer detection system (Bond Polymer Refine; Vision BioSystems) was used to reduce nonspecific staining. Tissue sections were treated with liquid DAB reagent; 3’-diaminobenzidine tetrahydrochloride was used as the chromogen, and hematoxylin was used as the counterstaining reagent. For analysis of total hnRNP K expression, specimens in which > 50% of the tumor cells displayed strong staining were defined as having ‘high-level total hnRNP K’ expression, and those in which ≤ 50% of tumor cells showed strong stained were defined as having ‘low-level total hnRNP K’ expression. For analysis of cytoplasmic hnRNP K, we used the method described previously [5], a sample was defined as ‘cytoplasmic positive’ in cases where >10% of the tumor cells exhibited cytoplasmic staining and as ‘cytoplasmic negative’ where ≤10% of cells were stained. For analysis of nuclear hnRNP K expression, specimens in which >50% of tumor cells displayed strong staining were defined as ‘high level of nuclear hnRNP K’ and those where ≤50% of tumor cells stained strongly were defined as ‘low level of nuclear hnRNP K.’ For analysis of MMP-12 expression, specimens in which > 20% of tumor cells displayed positive staining were defined as having ‘high-level’ MMP-12 expression, and those in which ≤ 20% tumor cells displayed positive staining were defined as having ‘low-level’ MMP-12 expression. MMP-12- and hnRNP K-positive tumor cells in representative microscopic fields were scored independently by two experienced pathologists.

### Western blotting

Whole cell lysates were prepared by incubating cells in NP40 lysis buffer (50 mM Tris–HCl, pH 7.5, 150 mM NaCl, 1% Igepal CA-630 and protease inhibitors [4.76 μg/ml leupeptin, 3.25 μg/ml aprotinin, 0.69 μg/ml pepstatin and 1 mM phenylmethylsulfonyl fluoride]) on ice for 30 min. The lysates were then centrifuged at 12,000 × *g* at 4°C for 10 min to pellet cell debris, and the supernatant was collected. In addition, the expression of MMP12 protein in the serum-free medium of NPC cells was measured by Western blotting. Conditioned media were collected and concentrated 20-fold using Amicon Ultra-4 centrifugal filters (Millipore, Carrigtwohill Co., Cork, Ireland) according to the manufacturer’s protocol. Protein concentration was determined using the Bradford reagent. Equal amounts of protein were resolved by electrophoresis on SDS-polyacrylamide gels, and the resolved proteins were transferred to nitrocellulose membranes. The membranes were blocked in 0.1% TBS-Tween 20 (TBST) with 5% non-fat dry milk for 1 h, and then incubated overnight with anti-hnRNP K (1:1000), anti-MMP12 (1:500), anti-PGK1(1:1000) (all from Santa Cruz Biotechnology, Santa Cruz, CA), and anti-actin (1:5000, MDBio, Piscataway, NJ). The membranes were then incubated with secondary antibodies coupled to horseradish peroxidase (1:5000), and the results were visualized using an enhanced chemiluminescence system (Amersham Pharmacia Biotech, AB, Uppsala, Sweden).

### Zymography

NPC cells treated with hnRNP K-targeting siRNA were cultured in serum-free medium for 48 h, and the conditioned medium was harvested and concentrated 20-fold using an Amicon Ultra-4 centrifugal filter (Millipore). The protein concentration was quantified using the Bradford reagent and protein was mixed with non-reducing sample buffer. The protein mixture was heated at 37°C for 30 min and separated by electrophoresis on an SDS-polyacrylamide gel containing 1 mg/ml α-casein. The gel was washed twice with 2.5% Triton X-100 for 30 min at room temperature (RT), and incubated in developing buffer (50 mM Tris–HCl, pH 7.6, 15 mM NaCl, 10 mM CaCl_2_ and 0.02% NaN3) for 15 min at RT with gentle agitation. The gel was then transferred to fresh developing buffer and incubated at 37°C for 48 h, and then incubated in fixing buffer for 15 min at RT with gentle agitation. The gel was stained with 0.125% Coomassie blue at RT for 1 hr and destained with fixing buffer; the solution was changed every 15 min until caseinolytic bands were visible. The caseinolytic band found at 54 kDa was subjected to zymographic measurement of MMP12 activity.

### Plasmid construction

The promoter sequences of human MMP12 were obtained from the UCSC genome browser. Using human genomic DNA isolated from normal peripheral blood mononuclear cells (PBMCs) as the template, we obtained the MMP12 promoter −2000 (−2000 to +97) fragment by PCR using the following primers: forward, 5'-TCCCC CGGGA CATAG AAAAA TTATC TAGTC CTACG TGTA-3' and reverse, 5'-CCGCT CGAGT GTAAA CTTCT AAACG GATCA ATTCA GTTT-3' (including SmaI and XhoI sites, respectively). The resulting PCR product was ligated into the SmaI and XhoI sites of the pGL3-basic vector (Promega, Madison, WI). To generate 5' serial deletions of the MMP 12 promoter, fragments −1500 (−1500 to +97), −999 (−999 to +97), −499 (−499 to +97), −399 (−399 to +97), −299 (−299 to +97), −199 (−199 to +97), −99 (−99 to +97), −42 (−42 to +97), −32 (−32 to +97) or +2 (+2 to +97) were amplified from pGL3-MMP12 -2000 and ligated into the SmaI/XhoI-treated pGL3-basic vector.

### Luciferase assay

NPC-TW02 cells in 24-well plates were co-transfected with 0.4 ng of pRL-TK (Promega) and 0.8 μg of pGL3-basic vector with or without MMP12 promoter fragments, using Lipofectamine (Invitrogen) according to the manufacturer’s instructions. After 24 hours, Firefly and Renilla luciferase activities were measured using the Dual-Glo Luciferase Assay System (Promega) according to the manufacturer's instructions. Firefly luciferase activities were normalized to Renilla activities. Each bar represents an average of at least three independent experiments, and the error bars show standard deviations calculated using Microsoft Office Excel.

### DNA pull-down assay

Probes corresponding to the potential binding elements within the MMP12 promoter were generated by PCR using the appropriate biotinylated primers, as follows: −42/+97 (−42 to +97) forward, 5'-GGGAT GATAT CAACT ATGAG TCACT CATAG G-3' and reverse, 5'-TGTAA ACTTC TAAAC GGATC AATTC AG-3'; and +2/+97 (+2 to +97) forward, 5'-AGAAC CCGGA CTAAG GGC-3' and reverse, 5'-TGTAA ACTTC TAAAC GGATC AATTC AG-3'. The biotinylated probes were conjugated with M-280 Streptavidin Dynabeads (Invitrogen) in binding buffer (10 mM Tris–HCl, pH 7.5, 50 mM KCl, 1 mM MgCl_2_, 1 mM EDTA, pH 8.0, 1 mM Na_3_VO_4_, 5 mM DTT, 5% glycerol, 0.3% NP-40) for 40 min at room temperature. NPC-TW02 cells were extracted using the Compartmental Protein Extraction Reagent (Millipore), and nuclear fractions (50 μg) were incubated with unconjugated Dynabeads in the presence of 25 μg/ml poly (dI:dC) for 20 min at RT. The unbound fraction was incubated with 250 μg of Dynabeads bound to 50 pmol of immobilized probe for 1 h at RT. The Dynabead-bound complexes were collected using a Dynal MPC-S magnetic particle concentrator (Dynal Biotech, Oslo, Norway) and washed with binding buffer. The DNA-bound proteins were eluted in SDS sample buffer and assayed by Western blotting.

### Chromatin immunoprecipitation (ChIP) assays

ChIP assays were performed using a Magna ChIP™ Kit (Millipore) according to the manufacturer’s protocol, with modifications. Briefly, NPC-TW02 cells (1×10^7^) were cross-linked by treatment with 1% formaldehyde for 10 min on ice, and the reactions were quenched with glycine (0.125 M) for 10 min on ice. Fixed cells were washed twice with ice-cold PBS and lysed for 15 min on ice with the provided cell lysis buffer and protease inhibitors. The samples were then centrifuged at 800 *x g* for 5 min at 4°C, the supernatants were removed, and the pellets were resuspend with the provided nuclear lysis buffer and protease inhibitors. Chromatin was sheared by sonication (eight times for 10 seconds each at 30% output) on ice and centrifuged at 10,000 × *g* for 10 min at 4°C. The supernatant was collected and diluted 10-fold with ChIP dilution buffer (0.01% SDS, 1.1% Triton X-100, 1.2 mM EDTA, 16.7 mM Tris–HCl, pH 8.1, 167 mM NaCl) containing protease inhibitors. The diluted samples were incubated overnight at 4°C with 4 μg of an anti-hnRNP K antibody (Invitrogen) and magnetic protein A/G beads (Millipore). Mouse IgG (Millipore) was used as a control antibody. The immunocomplexes were collected using a Dynal MPC-S magnetic particle concentrator (Dynal Biotech) and washed once each in low-salt buffer, high-salt buffer, LiCl buffer, and Tris-EDTA buffer. The samples were resuspended in ChIP elution buffer (200 mM NaCl, 1% SDS, 20 mM Tris–HCl, pH 7.4) containing 100 μg/ml proteinase K, incubated for 2 h at 62°C, and then incubated for 10 min at 95°C. The DNA fragments were further purified using a QIAquick PCR Purification Kit (QIAGEN, Hilden, Germany), and quantitative PCR was performed using primers against the potential hnRNP K-binding elements in the MMP12 promoter.

### Lentiviral production and transduction

The negative control shRNA (lacZ) and two shRNAs targeting different sequences of human MMP12 (KD1, clone ID TRCN0000050209; and KD2, clone ID TRCN0000372998) in the pLKO.1-puro vector backbone were purchased from the National RNAi Core Facility of Academia Sinica (Taipei, Taiwan). For lentiviral production, 293 T cells were seeded at 4 × 10^5^/well in 6 well-plates and transfected with 1.8 μg pCMV-Δ8.91, 0.2 μg pMD.G and 2 μg lentiviral vector. Six hours after transfection, the culture medium was change to DMEM supplemented with 1% FCS. Supernatants were collected at 24 and 48 h after transfection, pooled, filtered through a 0.22-μm filter, and frozen at −80°C until use. For lentiviral transduction, NPC-TW02 cells were seeded at 2 × 10^5^/well in 6-well plates and infected with lentivirus in the presence of 8 μg/mL of polybrene. The transduced cells were selected with 1 μg/ml puromycin for 2–3 weeks.

### Cell proliferation assay

Equal numbers of MMP12-knockdown cell clones were dispensed to 6-well plates, and total cell numbers were counted on days 1, 2, 3 and 4 after plating. The results are presented as the mean ± SD from four independent experiments.

### Cell migration and invasion assays

The migration and invasion of NPC cells were evaluated using Transwell inserts (Corning Inc., Corning, NY, USA) and Biocoat Matrigel invasion chambers (BD Biosciences, Bedford, MA), respectively. For cell migration assays, the cells were washed twice with serum-free medium and resuspended in serum-free medium, and 1.8 × 10^5^ cells in 0.1 ml were added to the upper chamber of the apparatus. The lower chamber contained 0.6 ml medium with 10% FBS. For cell invasion assays, the same procedures were used, except that 2.5 × 10^5^ cells were resuspended in 0.5 ml of serum-free medium, and added to the upper chamber of the apparatus, while the lower chamber contained 0.75 ml medium with 10% FBS. After 24 h at 37°C, the migrated and invading cells were fixed and stained for 20 min with 0.25% crystal violet, 10% formaldehyde and 80% methanol, and the filters were washed five times with ddH_2_O to remove non-adherent cells. Ten to fifteen random fields (× 100 magnification) were captured for each membrane. The migrated or invading cells were counted and averages were calculated; results were obtained from three independent experiments. The relative fold-change in the number of migrated or invasive cells is shown, with the results from control cells given as 1.0. The effect of MMP12-specific inhibitor PF-356231 (Enzo Life Science, Farmingdale, NY) [41,42] on the migration of NPC cells was determined after culturing for 24 h in the presence of indicated concentrations of inhibitor or DMSO (as vehicle control). The invasive activities of NPC cells were determined after 24 h (NPC-TW02) or 36 h (NPC-HK1) of treatment with inhibitor.

### Statistical analysis

All statistical analyses were performed using the SPSS 13.0 statistical software package. The relationship between MMP12 expression and hnRNP K expression was evaluated using the Pearson Chi-Square test. *In vitro* data were analyzed with the Student’s *t* test. Differences were considered significant at a level of *P* < 0.05.

## Results

### Systematic analysis of hnRNP K-regulated MMPs genes

**Figure 1:**
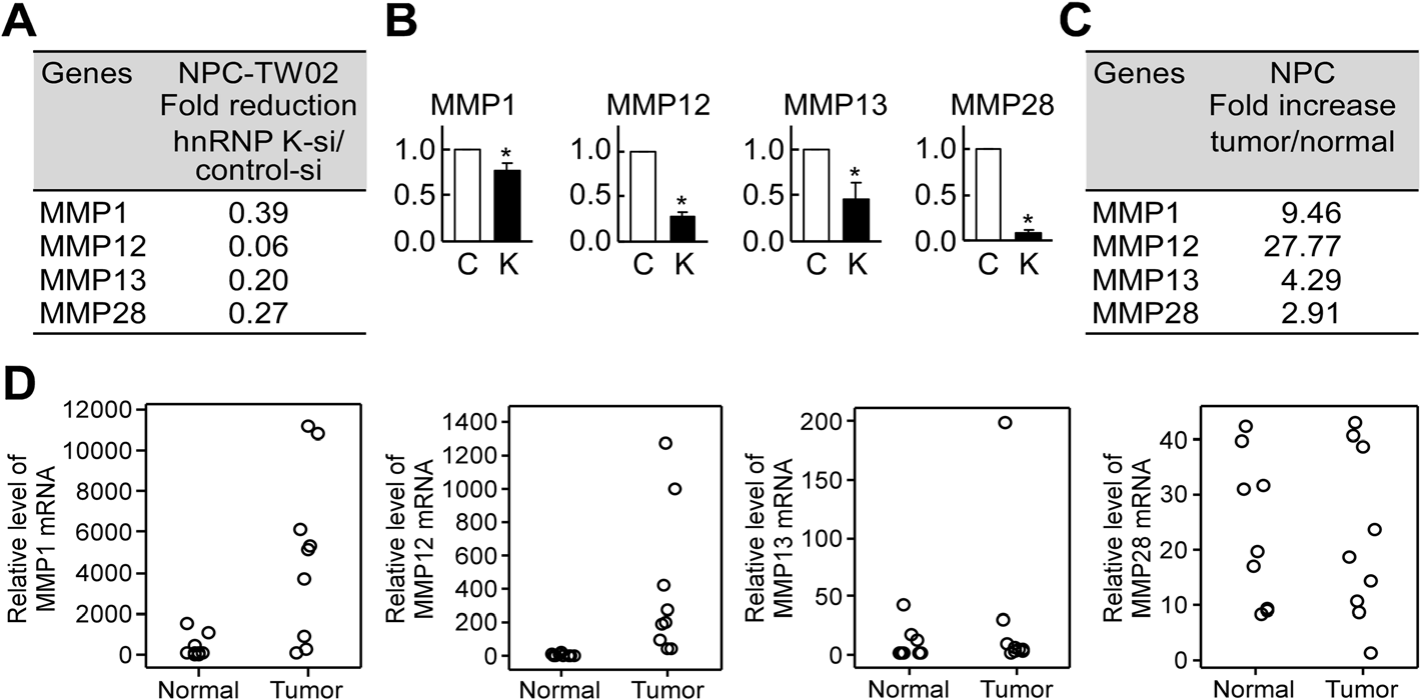
**Identification of hnRNP K-targeted MMPs. (A)** Affymetrix cDNA microarrays were used to examine the gene expression levels of MMP1, MMP12, MMP13 and MMP28 in hnRNP K-knockdown versus control siRNA-transfected NPC-TW02 cells. **(B)** Verification of hnRNP K-regulated MMP gene expression in NPC cells. NPC-TW02 cells were transfected with either hnRNP K-targeting (K) or control (C) siRNA. Cells were harvested post-transfection 48 h to detect the MMPs mRNA expression levels by quantitative RT-PCR analyses. All data are presented as the mean ± SD from three experiments. *P < 0.05. **(C)** Affymetrix cDNA microarrays were used to examine the gene expression levels of MMP1, MMP12, MMP13 and MMP28 in NPC tumor versus adjacent normal tissues. **(D)** Quantitative RT-PCR analyses of MMP gene expression in nine matched-pairs of NPC tumor and adjacent normal tissues.

We previously showed that hnRNP K contributes to the metastasis of NPC cells in part by regulating downstream genes [7]. Since the MMP family proteins are well known to be involved in tumor metastasis, we tested if they could be regulated through hnRNP K. We used Affymetrix cDNA microarrays to compare the expression profiles of MMP family genes in NPC-TW02 cells transiently transfected with hnRNP K-targeting siRNA versus those transfected with negative control siRNA, and in NPC tissue samples and adjacent normal tissues. The 7 out of 23 MMP genes showed reduced expression (1.5-fold decrease) in hnRNP K-knockdown cells (Additional file 2: Table S2), while 11 out of 23 were elevated (1.5-fold increase) in NPC tissues (Additional file 3: Table S3). Among these differentially expressed genes, MMP1, MMP12, MMP13 and MMP28 were consistently reduced in hnRNP K-knockdown cells (Figure 1A) but elevated in tumor cells (Figure 1C). We further confirmed our microarray results using quantitative RT-PCR, and found that the mRNA levels of MMP1, MMP12, MMP13 and MMP28 were significantly reduced (to 0.77-, 0.28-, 0.46- and 0.09-fold, respectively; *P* < 0.05) in hnRNP K-knockdown cells compared with control siRNA-treated NPC-TW02 cells (Figure 1B). On the other hand, the mRNA levels of MMP1 and MMP12 were significantly elevated (14.2- and 56.3-fold, respectively; P < 0.05) in nine matched-pairs of NPC tumor and adjacent normal tissues. NPC tumor samples compared with adjacent normal tissues, whereas the mRNA levels of MMP13 and MMP28 were not significantly different between the tumor and adjacent normal tissues (Figure 1D). As MMP12 has not previously been examined in the context of NPC, it was chosen for further study.

### Correlation of MMP12 and hnRNP K expression levels in NPC tissues

**Figure 2:**
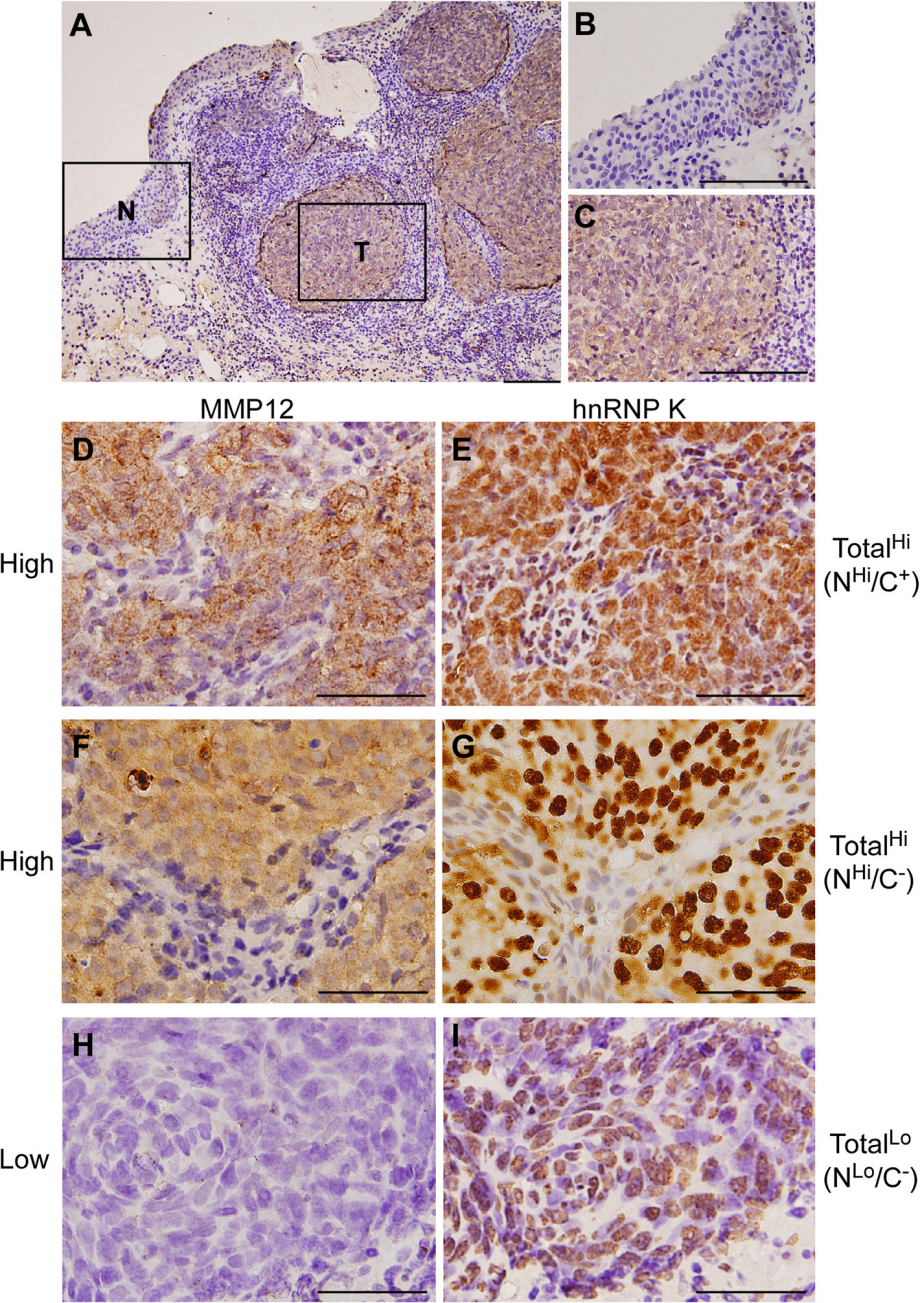
**Correlation of MMP12 and hnRNP K expression in NPC biopsy tissues. (A)** Immunohistochemical staining of MMP12. A representative NPC biopsy tissue sample containing adjacent nontumor (N) and tumor (T) cells stained with a specific MMP12 antibody is shown at 200x magnification. **(B and C)** The N and T areas are shown at 400x magnification, respectively. **(D-I)** Consecutive NPC tissue sections were stained using anti-hnRNP K and anti-MMP12 antibodies, and subjected to immunohistochemical assessment. Representative NPC tissue sections with high-level expression of MMP12 **(D and F)** and total hnRNP K (**E**, N^Hi^/C^+^; **G**, N^Hi^/C^−^) and low-level expression of MMP12 **(H)** and hnRNP K (**I**, N^Lo^/C^−^) are shown. Total^Hi^, total hnRNP K high; Total^Lo^, total hnRNP K low; N^Hi^, nuclear hnRNP K high; N^Lo^, nuclear hnRNP K low; C^+^, cytoplasmic hnRNP K positive; C^−^, cytoplasmic hnRNP K negative; scale bar in **(A-C)** = 100 μm, **(D-I)** = 50 μm.

The epithelial-stromal cell cross contamination is known to be one of problems in the analysis of RNA/protein expression from solid tumor. Therefore, 82 NPC biopsy specimens were subjected to immunohistochemical (IHC) analysis and the differential expression of MMP12 and hnRNP K between the tumor and normal epithelial tissues were investigated. Patient characteristics and clinical features are summarized in Table 1. In general, our IHC data demonstrated that the NPC tumor cells expressed higher levels of MMP12 compared to adjacent normal cells. As shown in Figure 2A-C, consecutive tissue slides of the same set of specimens were used to evaluate the protein expression levels of MMP12 and hnRNP K. We further analyzed whether the expression level of MMP12 correlated with the subcellular localization (cytoplasmic or nuclear) of hnRNP K in NPC cells. We assessed the association between MMP12 expression (high or low) and (1) the total hnRNP K expression (high or low), or (2) the nuclear hnRNP K expression (high or low), or (3) the cytoplasmic hnRNP K expression (positive or negative). The statistical analysis was summarized in Table 2. Statistical analyses revealed that high-level MMP12 expression was significantly correlated with high-level of total hnRNP K (*P* = 0.026; Table 2) and nuclear hnRNP K (*P* = 0.042; Table 2), rather than cytoplasmic hnRNP K (*P* = 0.168; Table 2). These results suggest that nuclear hnRNP K was positively correlated with MMP12 in NPC tumor cells.

**Table 1:** Characteristics of 82 patients with nasopharyngeal carcinoma

**Characteristics**	**Subject number**
Age (years)	
mean ± SD	46.8 ± 13.1
Gender	
Male	61
Female	21
T stage	
1-2	52
3-4	30
N stage	
0-1	50
2-3	32
Clinical stage	
I-II	17
III-IV	65
Histological type	
Keratinizing carcinoma	4
Non-keratinizing carcinoma	
Undifferentiated subtype	69
Differentiated subtype	8
Basaloid squamous cell carcinoma	1
Total	82

**Table 2:** Correlation of hnRNP K and MMP12 expression

	**MMP12 expression**		***P***
	**High**	**Low**	
Total hnRNP K			0.026*
High	32	15	
Low	15	20	
Nuclear hnRNP K			0.042*
High	25	10	
Low	22	25	
Cytoplasmic hnRNP K			0.168
Positive	33	19	
Negative	14	16	

*With statistic significance.

### The expression and activity levels of MMP12 are regulated by hnRNP K in NPC cells

**Figure 3:**
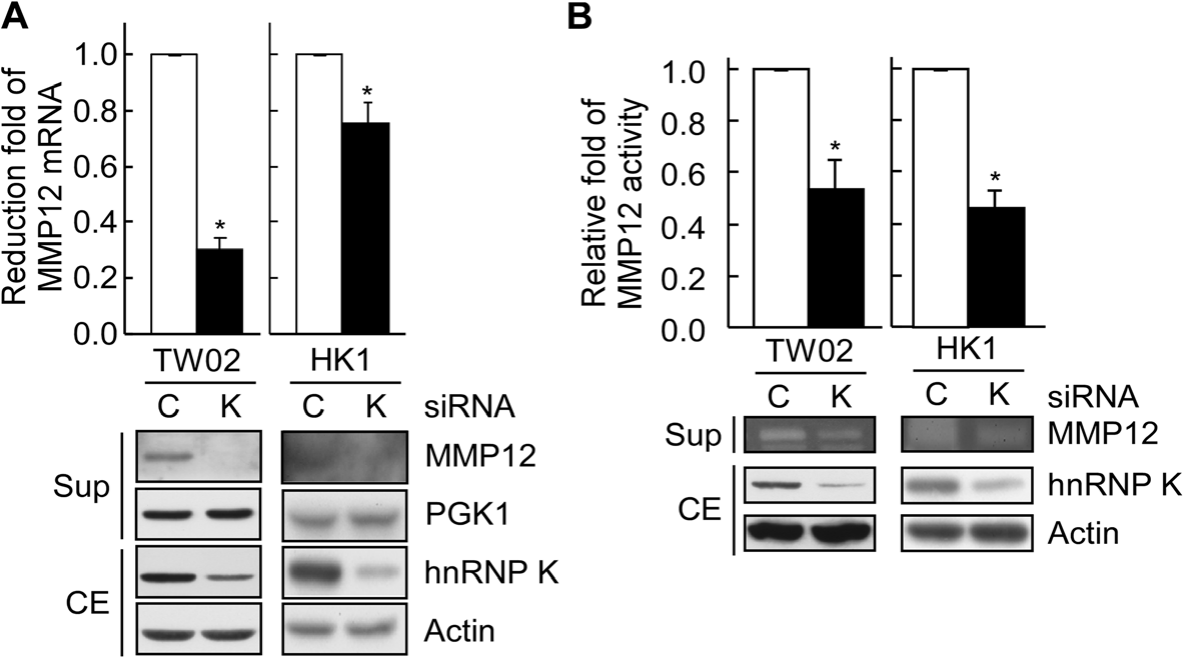
**Suppression of hnRNP K expression downregulates the expression and activity of MMP12 in NPC. (A)** NPC-TW02 and -HK1 cells were transfected with hnRNP K-targeting (K) or control (C) siRNA. Twenty-four hours after transfection, cells were further cultured in serum-free medium for another 48 h. MMP12 mRNA levels were determined by quantitative RT-PCR, and MMP12 and hnRNP K protein levels in the culture supernatant (Sup) and in the cell extract (CE), respectively, were examined by Western blotting. PGK1 and Actin protein levels were used as the loading control for the secreted and the cytoplasmic proteins, respectively. **(B)** The enzymatic activity of MMP12 was analyzed by zymography. The supernatants from the NPC cells treated with either hnRNP K-targeting (K) or control (C) siRNA were collected after 48 h, and were subjected to zymographic analysis. The protein levels of hnRNP K and actin in the cell extracts were analyzed by Western blotting.

To gain insight into the potential role of hnRNP K in regulating MMP12 expression, we tested MMP12 expression in hnRNP K-knockdown and control cells of two NPC cell lines (NPC-TW02 and -HK1). As shown in Figure 3A, the level of MMP12 mRNA was reduced significantly in hnRNP K siRNA-treated NPC cells compared with control siRNA-treated cells. To assess whether the effect of hnRNP K knockdown on MMP-12 mRNA was correlated with changes in the protein and/or enzymatic levels, we performed Western blot and zymographic analyses. Conditioned media were obtained from the above-described NPC cells and analyzed by immunoblotting and casein zymography. Western blot analysis and casein zymography showed that hnRNP K knockdown significantly reduced the protein expression and activity levels of MMP12 (54 kDa), respectively (Figure 3A and B). Thus, hnRNP K knockdown inhibited the mRNA expression, protein expression and enzymatic activity of MMP12.

### MMP12 is transcriptionally regulated by hnRNP K

**Figure 4:**
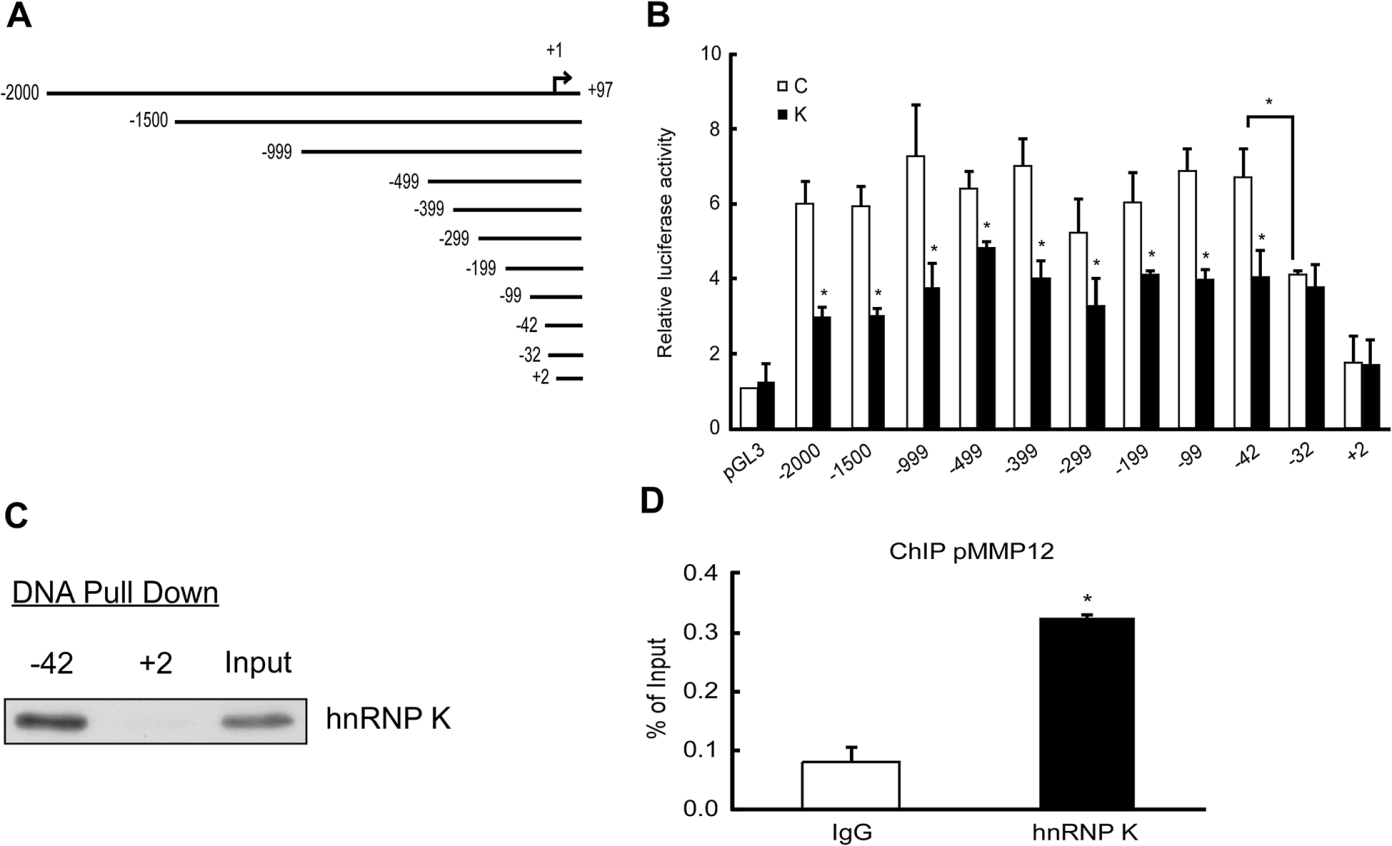
**Transcriptional regulation of MMP12 by hnRNP K. (A)** Schematic diagrams of the utilized reporter constructs, which contained 5' serial deletions of the MMP12 promoter. **(B)** NPC-TW02 cells were pretreated with control siRNA (C) or hnRNP K-targeting siRNA (K) for 24 h and then transfected with pGL3-basic (pGL3) with or without 5' serial deletions of the promoter sequence of the MMP 12. Firefly and Renilla luciferase activities were determined at 24 h post-transfection. *P < 0.05. **(C)** DNA pull-down assays were performed using nuclear extracts isolated from NPC-TW02 cells and 5' biotin-labeled probes corresponding to the −42/+97 (−42 to +97) or +2/+97 (+2 to +97) regions of the MMP12 promoter. The hnRNP K levels in the immunoprecipitates and 1% inputs were determined by Western blotting. **(D)** Chromatin immunoprecipitation was carried out using nuclear extracts from NPC-TW02 cells and an antibody against hnRNP K, followed by quantitative PCR of a sequence within the MMP12 promoter region (−95 to −20). Mouse IgG immunoprecipitation was done as a negative control. *P < 0.01.

We further clarified the mechanism(s) underlying the hnRNP K-mediated regulation of MMP12 expression. To discriminate between transcriptional activation and post-transcriptional regulation, we analyzed the effect of hnRNP K knockdown on MMP12 promoter activity and mRNA stability. As shown in Figure 4A and B, NPC-TW02 cells were treated with siRNA followed by transfection of constructs containing 5' serial deletions of the MMP12 promoter, and reporter activity was examined 24 h later. Our results revealed that knockdown of hnRNP K significantly inhibited the activity of MMP12 promoter constructs containing the deletion from −2000 to −42 bp of the transcription start site. (*P* < 0.05). There had no effect on MMP12 promoter (−32 to +97) while cells treated with hnRNP K siRNA compared with control group (Figure 4B). Moreover, the MMP12 promoter construct spanning −32 to +97 showed substantially less activity compared with that spanning −42 to +97 (61.3% vs. 100%, respectively) (Figure 4B). These results collectively suggest that the MMP12 promoter region covering −42 to −33 may be the potential hnRNP K response region. To further verify the binding of hnRNP K to the MMP12 promoter, we performed *in vitro* DNA pull-down assays with probes spanning −42 to +97 and +2 to +97 of the MMP12 promoter. As shown in Figure 4C, hnRNP K specifically bound to probe (−42 to +97) but not probe (+2 to +97), suggesting that the −42 to +1 region is indispensable for hnRNP K binding. To further support our contention that hnRNP K can interact with the endogenous MMP12 promoter, we performed a chromatin-immunoprecipitation analysis. As shown in Figure 4D, hnRNP K specifically immunoprecipitated with the MMP12 promoter. Together, these results indicated that the hnRNP K-responsive region is the sequence of −42 to −33 bp upstream of the MMP12 transcription start site.

In addition, we examined the effect of hnRNP K knockdown on MMP12 mRNA stability. Treatment of NPC-TW02 cells with actinomycin D to block *de novo* RNA synthesis, and used quantitative RT-PCR to examine MMP12 mRNA levels at 2, 4, 8, 12 and 16 h post-treatment. The half-life of the MMP12 mRNA was 31.07 h in hnRNP K-knockdown cells and 38.17 h in control cells, which was not significantly different (Additional file 4: Figure S1). Taken together, our findings indicate that the hnRNP K-mediated changes in MMP12 gene expression arise via promoter inhibition, not mRNA destabilization.

### MMP12 promotes NPC cell migration and invasion

**Figure 5:**
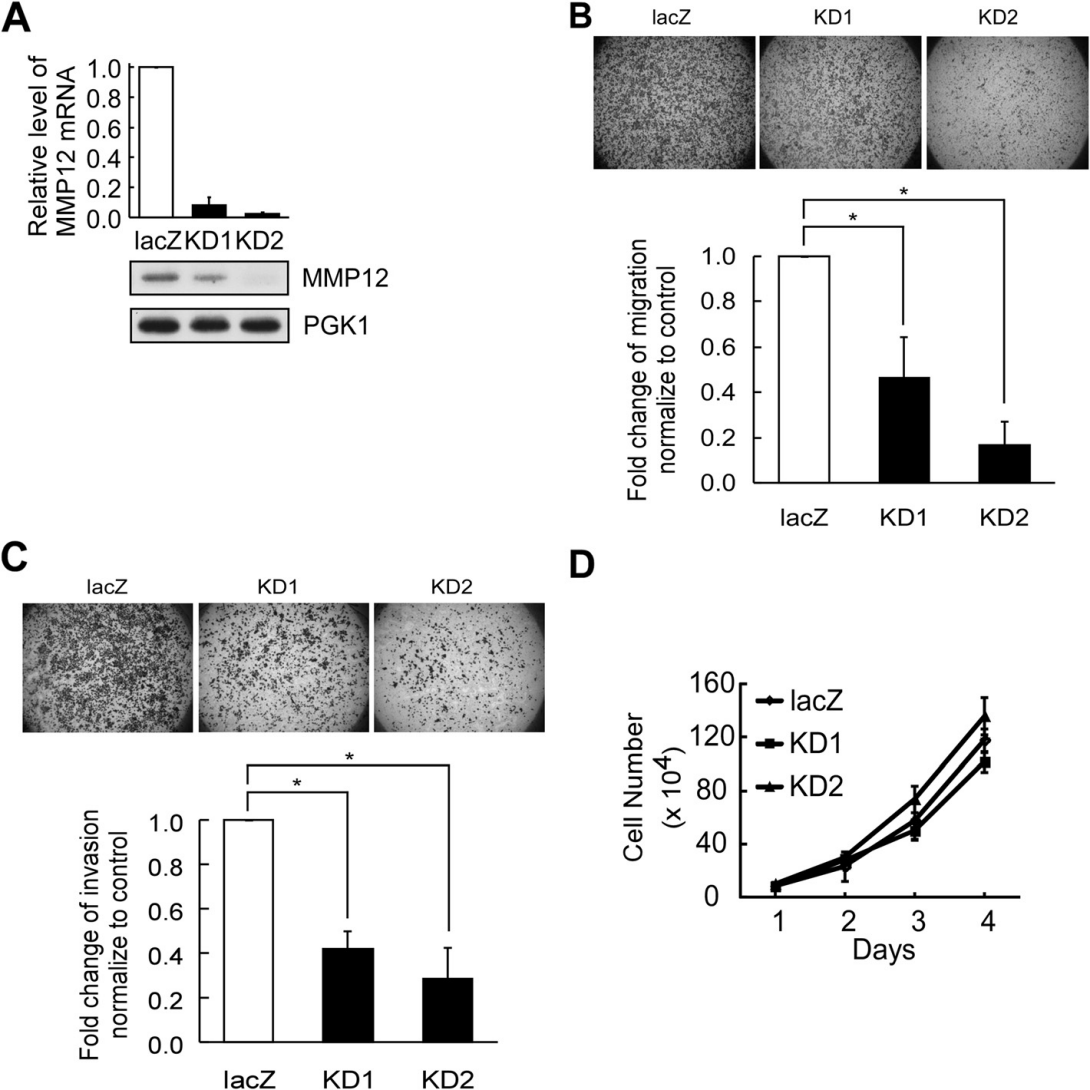
**MMP12 promotes cell migration and invasion in NPC-TW02 cells. (A)** MMP12 mRNA expression was detected in stable NPC-TW02 cells of MMP12-knockdown (KD1 and KD2) and control (LacZ) by quantitative RT-PCR, and MMP12 protein levels in the culture supernatant were examined by Western blotting. PGK1 protein levels were used as the loading control for secreted proteins. Cell migration **(B)** and invasion **(C)** assays were performed in stable NPC-TW02 cells of MMP12-knockdown (KD1 and KD2) and control (LacZ). Images were captured at 24 h under 12.5× magnification. The relative fold-change in the number of migrated cells is shown, with the results from control cells given as 1.0. All data are presented as the mean ± SD from three independent experiments. *P < 0.05. **(D)** Cell growth in stable NPC-TW02 cells of MMP12-knockdown (KD1 and KD2) and control (LacZ) was determined by counting the cell numbers for 4 days.

**Figure 6:**
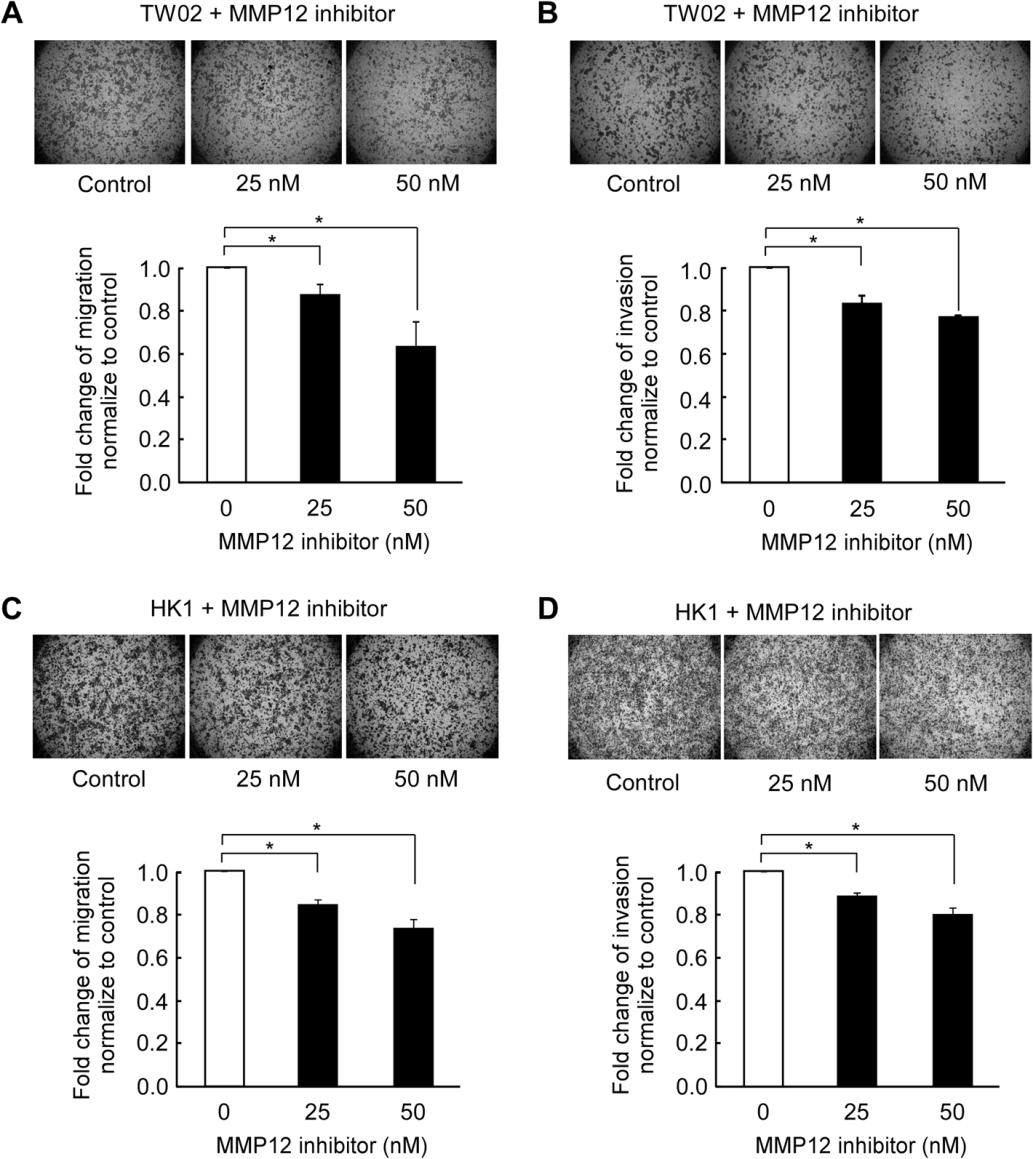
**MMP12-specific inhibitor, PF-356231, inhibits cell migration and invasion in NPC cells in a dose-dependent manner.** Cell migration and invasion assays were performed in NPC-TW02 **(A and B)** and in NPC-HK1 **(C and D)** cells, respectively, in the presence of different concentration of MMP12 inhibitor PF-356231 (0, 25 and 50 nM). Images were captured under 12.5x magnification. The relative fold-change in the number of migrated cells is shown, with the results from control cells given as 1.0. All data are presented as the mean ± SD from three independent experiments. *P < 0.05.

To examine the biological function of MMP12 in NPC cells, we established two MMP12-knockdown cell lines using lentiviral transduction of two different MMP12-targeting shRNA sequences. As shown in Figure 5A, the MMP12 protein and mRNA levels were reduced in the two MMP12-knockdown cell lines compared to control cells transduced with a control shRNA targeting LacZ. Importantly, cell migration (Figure 5B) and invasion (Figure 5C) were significantly and dose-dependently reduced in the MMP12-knockdown cells compared to controls (*P* < 0.05). However, the reduction of migration and invasion in MMP12-knockdown cells were not due to the difference in cell growth (Figure 5D) between MMP12-knockdown and control cells. We further investigated the effect of the treatment of PF-356231, a specific inhibitor of MMP12 on the migration and invasion of NPC cells. As compared to untreated control, PF-356231 treatment significantly and dose-dependently reduced the migration (Figure 6A) and invasion (Figure 6B) in NPC-TW02 cells (*P* < 0.05). Similar results were observed in NPC-HK1 cells (Figure 6C and D). Taken together, these results indicate that hnRNP K-mediated MMP12 expression enhances the migration and invasion of NPC cells. In addition, MMP12-mediated cell migration and invasion can be inhibited by PF-356231 treatment.

## Discussion

Overexpression of hnRNP K has been found in various cancers and correlates with poor prognosis [3-5,43]. Here, we report a new function for hnRNP K-regulating MMP12, which can induce cell migration and invasion in NPC cells. We further show that the sequence −42 to −33 bp relative to the transcription start site of MMP12 is bound by hnRNP K, triggering the transcriptional activation of MMP12. Moreover, MMP12 promotes cell migration and invasion in NPC cells, and high-level MMP12 expression was found to be correlated with increased expression of hnRNP K in NPC patients. Collectively, our findings show that hnRNP K binds the MMP12 promoter, thereby inducing MMP12 expression through transcriptional activation. This provides a mechanistic explanation for the correlation of hnRNP K with MMP12 and metastasis in NPC. Although we and other groups have showed that an aberrant cytoplasmic localization of hnRNP K was correlated with a poor prognosis in many tumors including NPC [3,5,44], in this study, we found that the nuclear but not the cytoplasmic hnRNP K is significantly correlated with MMP12 expression level. Conceivably, only the nuclear hnRNP K can transcriptionally regulate the MMP12 gene expression. On the contrary, TP, a hnRNP K target gene, whose expression is upregulated through the increase in its mRNA stability by the binding of cytoplasmic hnRNP K [11]. From these data, we can conclude that hnRNP K has dual roles in different subcellular localization. Whether nuclear or cytoplasmic hnRNP K is responsible for regulating its downstream target genes, it depends largely on the target gene itself.

HnRNP K overexpression has been correlated with poor distant metastasis-free survival [5,45-47], suggesting that hnRNP K can promote tumor metastasis. However, the underlying mechanism responsible for this promotion of metastasis was previously unknown. In the present study, our systematically analysis of the MMP gene family revealed that MMP12 was induced by hnRNP K and could promote cell migration and invasion in NPC cells. Importantly, high-level MMP12 expression was correlated with increased expression of hnRNP K in NPC patients, suggesting that MMP12 is at least partially responsible for the hnRNP K-mediated metastasis of NPC. Consistent with our hypothesis, elevated expression of MMP12 was previously associated with metastatic disease in non-small cell lung cancer [27] and head and neck squamous cell carcinoma [25].

Activities of MMPs are linked to many metastasis-associated events in cancer progression. Therefore, MMPs may be the ideal targets for anti-cancer drug discovery. The partial inhibition of cell migration and invasion was observed after MMP12 inhibitor PF-356231 treatment (Figure 6), implying that there are multiple pathways, besides MMP12, may involve in promoting cell motility in NPC. For instance, AP-1-mediated MMP3 activation [48], NF-κB-mediated MMP9 activation [22], JNK/AP-1/DNMT/E-cadherin silencing [49] and downregulation of microRNA-144- mediated PTEN activation [50], these pathways have been reported to promote migration ability in NPC. Therefore, hnRNP K-mediated activation of MMP12 may partly contribute to enhance NPC cell migration. In addition, recent work has shown that forced overexpression of hnRNP K can increase the invasive capacity of mouse fibroblasts NIH3T3 by increasing MMP3 expression [45], although the expression level of MMP3 was not changed in hnRNP K-knockdown human NPC cells (Additional file 2: Table S2). Taken together, the previous findings and our present results indicate that hnRNP K may promote tumor metastasis by modulating the ECM via MMP induction. In addition, PF-356231 may be considered to treat NPC metastasis with high MMP12 expression.

The MMPs are involved in many phases of cancer progression, including tumor invasion, metastasis, and angiogenesis [16,17]. In addition to MMP12 (present study), MMP1 [51], MMP13 [52,53] and MMP28 [54] have also been shown to promote invasion and metastasis in various cancers. Importantly, hnRNP K can induce the expression of MMP1, MMP12, MMP13 and MMP28 in NPC cells (Figure 1B) and the expression of MMP3 in fibroblasts [45], suggesting that hnRNP K controls the expression levels of various MMPs. In addition to its effects on tumor metastasis, hnRNP K can contribute to tumor progression and malignancy through its antiapoptotic function [7,11]. Thus, the results of the present and previous studies collectively suggest that hnRNP K should be considered a potential target for the future development of new anticancer agents.

Previous studies have demonstrated that MMP2 and MMP9 expression can be induced in EBV-infected NPC cells [55]. Furthermore, it has been reported that the response of NPC cells to EBV infection is mediated mainly by the NF-κB and STAT3 signal cascades [56]. EBV infection has been known to lead to NPC tumorigenesis. And LMP1 is the most important viral oncoprotein that alters many cellular gene expression e.g. MMP2 and MMP9. We speculate that MMP induction initially required EBV infection and LMP1 expression, however, once the cells become NPC tumor cells, the presence of EBV or LMP1 is probably less important.

Although hnRNP K can regulate gene expression by binding to DNA and RNA [6,57], we found that it induces MMP12 mRNA expression by activating the MMP12 promoter rather than stabilizing the MMP12 mRNA (Additional file 4: Figure S1). Similar to the transcriptional induction of MMP12 by AP-1 [29,30], NFκB [29], β-catenin [58,59], YB-1 [60] and PPARα agonist [61], we herein show that hnRNP K can induce MMP12 expression through its association with the sequence −42 to −33 bp upstream of the MMP12 transcription start site. Previous studies showed that hnRNP K can regulate promoter activity by interacting with DNA-bound transcriptional activators [7,62]. The −42 to −33 bp region is close to an AP-1-responsive element at −26 to −19 [30], suggesting that future studies are warranted to examine the potential interaction of hnRNP K and AP-1.

## Conclusions

We herein show that hnRNP K exerts a metastatic function by inducing MMP12 via its binding to the −42 to −33 bp region of the MMP12 promoter, which controls transcriptional activation. MMP12 is overexpressed in NPC, and its expression is correlated with that of hnRNP K in NPC patients. Moreover, NPC metastasis with high MMP12 expression may be treated with MMP12-specific inhibitor, PF-356231. Based on these novel findings, we propose that hnRNP K and MMP12 should be considered as potential targets for the development of new anticancer agents.

## Abbreviations

NPC: Nasopharyngeal carcinoma; hnRNP K: Heterogeneous nuclear ribonucleoprotein K; MMP12: Matrix metalloproteinase 12; LMP1: Latent membrane protein 1; EBV: Epstein-Barr virus; ECM: Extracellular matrix; ChIP assay: Chromatin immunoprecipitation assays; DMEM: Dulbecco’s modified Eagle’s medium; FCS: Fetal calf serum; M-MLV: Moloney Murine Leukemia Virus; WHO: World Health Organization; TBST: TBS-Tween 20; RT: Room temperature; PBMCs: Peripheral blood mononuclear cells.

## Competing interests

The authors declare that they have no competing interests.

## Authors' contributions

ICC, LCC and YSC designed this study; ICC, LCC, AKC, MC, HYH, CH and YL performed the experiments; NMT and KPC oversaw collection of clinical samples; CH evaluated human nasopharyngeal carcinoma tissue sections; YL supervised histological staining and analysis; ICC, LCC and YSC evaluated the data, and ICC, LCC, HPL and YSC wrote the manuscript; YSC and HPL supervised the project. All authors read and approved the final manuscript.

## Pre-publication history

The pre-publication history for this paper can be accessed here:

http://www.biomedcentral.com/1471-2407/14/348/prepub

## Supplementary Material

Additional file 1: Table S1 Sequence of quantitative PCR Primers. Click here for file

Additional file 2: Table S2 Gene expression profiles of various MMPs in hnRNP K knockdown NPC-TW02 cell line. Click here for file

Additional file 3: Table S3 Gene expression profiles of various MMPs in NPC. Click here for file

Additional file 4: Figure S1 Effect of hnRNP K knockdown on the half-life of MMP12 mRNA. The levels of MMP12 mRNA in NPC-TW02 cells transfected with control siRNA (C) or hnRNP K siRNA (K) for 48 h were measured following treatment with actinomycin D for 2, 4, 8, 12 and 16 h. Click here for file
