# Supplementary material for: Matrix metalloproteinase 12 is induced by heterogeneous nuclear ribonucleoprotein K and promotes migration and invasion in nasopharyngeal carcinoma
Source: BMC Cancer. 2014 May 20;14:348. doi: 10.1186/1471-2407-14-348 (PMC4033617; doi:10.1186/1471-2407-14-348)
Supplement: Additional file 1: Table S1 — Sequence of quantitative PCR Primers. [file 1471-2407-14-348-S1.pdf]

Table S1. Sequence of quantitative PCR Primers

Primer	Sense Sequence
MMP1	TGGACCTGGAGGAAATCTTGC
	AGAGTCCAAGAGAATGGCCGA
MMP12	CGGGCCTAAAATTGATGCA
	GGTGATACGTTGGAGTAGGAAGTCA
MMP13	TGTTTCTTGTTGCTGCG
	TGTTTAGGGTTGGGGTC
MMP28	CCGTCCACTGCAGGAAAGAT
	CATCGACCCCCTTTGAAGAAG
GAPDH	TGGTATCGTGGAAGGACTCATGAC
	ATGCCAGTGAGCTTCCCGTTCAGC
