# Supplementary material for: Matrix metalloproteinase 12 is induced by heterogeneous nuclear ribonucleoprotein K and promotes migration and invasion in nasopharyngeal carcinoma
Source: BMC Cancer. 2014 May 20;14:348. doi: 10.1186/1471-2407-14-348 (PMC4033617; doi:10.1186/1471-2407-14-348)
Supplement: Additional file 2: Table S2 — Gene expression profiles of various MMPs in hnRNP K knockdown NPC-TW02 cell line. [file 1471-2407-14-348-S2.pdf]

Table S2. Gene expression profiles of various MMPs in hnRNP K knockdown NPC-TW02 cell line

**Genes**	**Probes**	**Fold change hnRNP K-si /control-si**
MMP1*	204475_at	0.385
MMP2*	201069_at	0.514
MMP3	205828_at	1.248
MMP7	204259_at	ND
MMP8	207329_at	ND
MMP9	203936_s_at	0.730
MMP10*	205680_at	0.340
MMP11	203878_s_at	ND
MMP12*	204580_at	0.057
MMP13*	205959_at	0.202
MMP14	160020_at	1.029
MMP15	243883_at	ND
MMP16*	207012_at	0.508
MMP17	206234_s_at	ND
MMP19	204575_s_at	ND
MMP20	207599_at	ND
MMP21	1552592_at	ND
MMP23B	207118_s_at	ND
MMP24	208387_s_at	ND
MMP25	207890_s_at	ND
MMP26	220541_at	ND
MMP27	220783_at	ND
MMP28*	219909_at	0.274

The relative fold-change of mRNA expression between NPC-TW02 treated with hnRNP K-si and control-si was determined by the mean of two independent Affymetrix microchip analyses. Affymetrix microarrays analysis has been previously described (Chen et al., 2010). Abbreviations and symbols: ND, not detected; *, 1.5-fold decrease in NPC-TW02 treated with hnRNP K-si compared with control-si.
