# Supplementary material for: Matrix metalloproteinase 12 is induced by heterogeneous nuclear ribonucleoprotein K and promotes migration and invasion in nasopharyngeal carcinoma
Source: BMC Cancer. 2014 May 20;14:348. doi: 10.1186/1471-2407-14-348 (PMC4033617; doi:10.1186/1471-2407-14-348)
Supplement: Additional file 3: Table S3 — Gene expression profiles of various MMPs in NPC. [file 1471-2407-14-348-S3.pdf]

Table S3. Gene expression profiles of various MMPs in NPC

**Genes**	**Probes**	**Fold change NPC/normal**
MMP1*	204475_at	9.464
MMP2	201069_at	1.056
MMP3*	205828_at	14.200
MMP7	204259_at	0.904
MMP8	207329_at	ND
MMP9*	203936_s_at	40.460
MMP10	205680_at	0.282
MMP11*	203878_s_at	4.708
MMP12*	204580_at	27.770
MMP13*	205959_at	4.293
MMP14*	160020_at	1.806
MMP15	243883_at	ND
MMP16	207012_at	0.351
MMP17	206234_s_at	ND
MMP19*	204575_s_at	2.625
MMP20	207599_at	ND
MMP21*	1552592_at	3.123
MMP23B	207118_s_at	ND
MMP24	208387_s_at	1.099
MMP25*	207890_s_at	1.755
MMP26	220541_at	0.228
MMP27	220783_at	ND
MMP28*	219909_at	2.911

The relative fold-change of mRNA expression between NPC and adjacent normal tissues was determined by Affymetrix microchip analysis. Affymetrix microarrays analysis has been previously described (Chen et al., 2010). Abbreviations and symbols: ND, not detected; *, 1.5-fold increase in NPC compared with normal tissues.
