# Supplementary material for: Matrix metalloproteinase 12 is induced by heterogeneous nuclear ribonucleoprotein K and promotes migration and invasion in nasopharyngeal carcinoma
Source: BMC Cancer. 2014 May 20;14:348. doi: 10.1186/1471-2407-14-348 (PMC4033617; doi:10.1186/1471-2407-14-348)
Supplement: Additional file 4: Figure S1 — Effect of hnRNP K knockdown on the half-life of MMP12 mRNA. The levels of MMP12 mRNA in NPC-TW02 cells transfected with control siRNA (C) or hnRNP K siRNA (K) for 48 h were measured following treatment with actinomycin D for 2, 4, 8, 12 and 16 h. [file 1471-2407-14-348-S4.pdf]

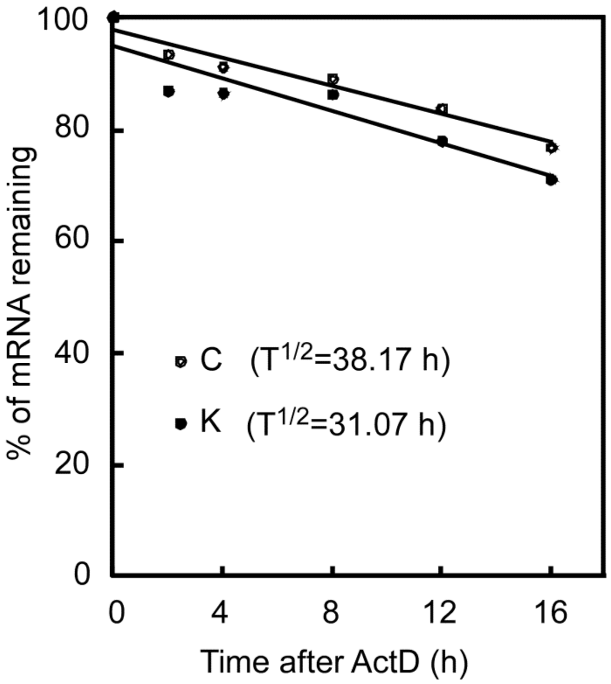

Figure S1 Effect of hnRNP K knockdown on the half-life of MMP12 mRNA. The levels of MMP12 mRNA in NPC-TW02 cells transfected with control siRNA (C) or hnRNP K siRNA (K) for 48 h were measured following treatment with actinomycin D for 2, 4, 8, 12 and 16 h.
